# Supplementary material for: QIIME2 enhances multi-amplicon sequencing data analysis: a standardized and validated open-source pipeline for comprehensive 16S rRNA gene profiling
Source: Microbiol Spectr. 2025 Jul 25;13(9):e01673-25. doi: 10.1128/spectrum.01673-25 (PMC12403817; doi:10.1128/spectrum.01673-25)
Supplement: Supplemental figures and tables — Fig. S1 to S3 and Tables S1 to S6. [file spectrum.01673-25-s0001.docx]

**Supplementary information**

# **QIIME2 enhances multi-amplicon sequencing data analysis: a standardized and validated open-source pipeline for comprehensive 16S rRNA gene profiling**

Armando G. Licata, Marica Zoppi, Chiara Dossena, Federico Rossignoli, Davide Rizzo, Manuela Marra, Giorgio Gargari, Giacomo Mantegazza, Simone Guglielmetti, Luca Bergamaschi, Olga Nigro, Stefano Chiaravalli, Maura Massimino, Loris De Cecco

| **Table of contents** |
| --- |
| 1. **Supplementary Figures**    1. Comparison of zymo mock community profiles using v2–9 region 16S rRNA gene analyses with greengenes and silva databases    2. Comparison of Sequencing Depth Across IR and QIIME 2 Pipelines    3. Comparative Procrustes Analysis of Paired Stool Samples Using Silva vs. Greengenes Databases at Genus and ASV Levels 2. **Supplementary tables**    1. Key functions to import QIIME2 data into R and calculate basic alpha and beta diversity metrics.    2. Pairwise post-hoc comparisons of microbial genera relative abundances across 16S rRNA regions in mock samples    3. Pairwise post-hoc comparisons of alpha diversity indices among sequencing methods and 16S rRNA regions    4. PERMANOVA results for microbial community structure across 16S rRNA regions in mock samples    5. Comparison of beta dispersion estimates for observed vs. theoretical mock community composition |
|  |

**1.1 Figure S1:** **Comparison of zymo mock community profiles using v2–9 region 16S rRNA gene analyses with greengenes and silva databases**

Stacked bar plots of relative abundances of bacterial genera present in the zymo mock community (leftmost bar) compared to replicate profiles generated with our V2–9 pipeline using either Greengenes v.13.5 (center) or Silva v.138-99 (right) as reference databases. Each color corresponds to a distinct genus. *Escherichia–Shigella* was initially undetected with Greengenes but identified in the Silva-based profiles at ~8.7% relative abundance.


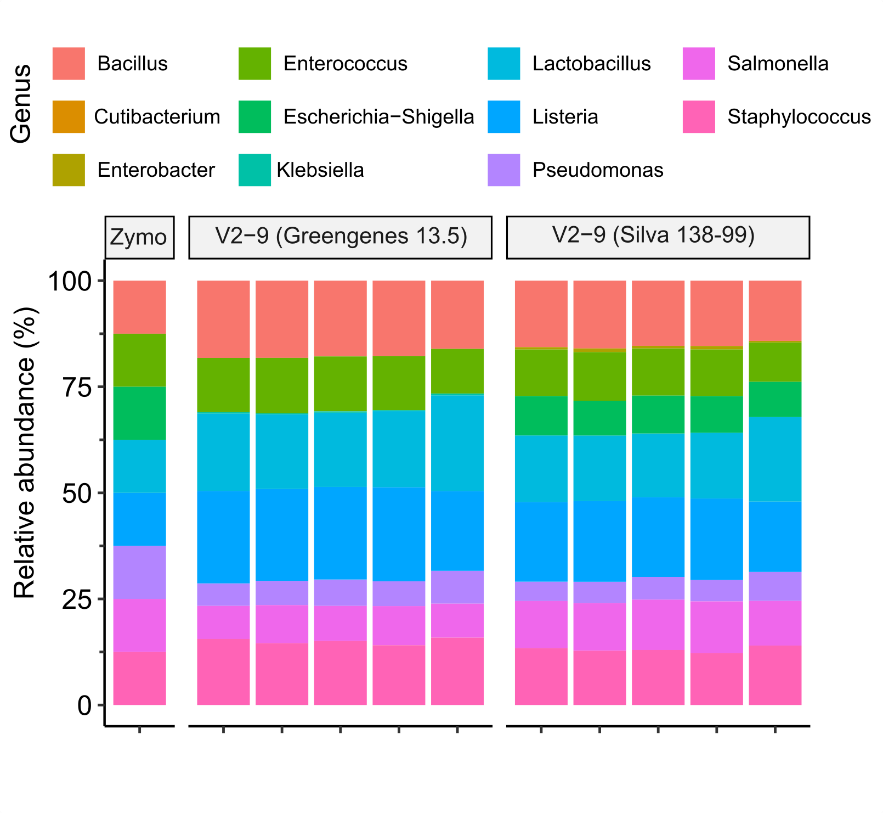


**1.2 Figure S2: Comparison of Sequencing Depth Across IR and QIIME 2 Pipelines**

Boxplot of log₂-transformed read counts across all 113 stool samples analyzed using Ion Reporter (IR) and QIIME 2 single- and multi-region pipelines. While the V2-9 pipeline achieved comparable sequencing depth to IR (ns), all single-region pipelines (V2, V3, V4, V6–7, V8, and V9) yielded significantly lower read counts (****P < 0.0001, Wilcoxon test).


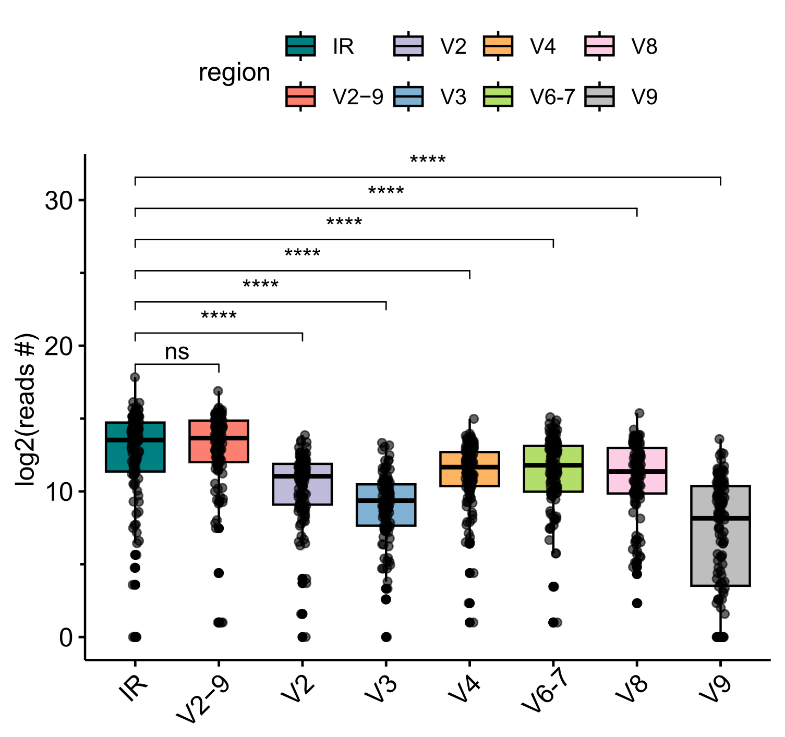


**1.3 Figure S3: Comparative Procrustes Analysis of Paired Stool Samples Using Silva vs. Greengenes Databases at Genus and ASV Levels**
Procrustes plots of stool‐microbiota profiles from the full INT-77/20 cohort (n = 113) processed in QIIME 2 (v.2023.7) with either the Greengenes (blue circles) or SILVA (red triangles) reference database at the Genus **(A)** and ASV **(B)** levels. Ordinations based on Bray–Curtis dissimilarities were superimposed to visualise concordance between databases; dashed grey lines join each sample’s Greengenes (v.13_5) and SILVA (v.138-99) coordinates, with shorter segments denoting closer agreement.


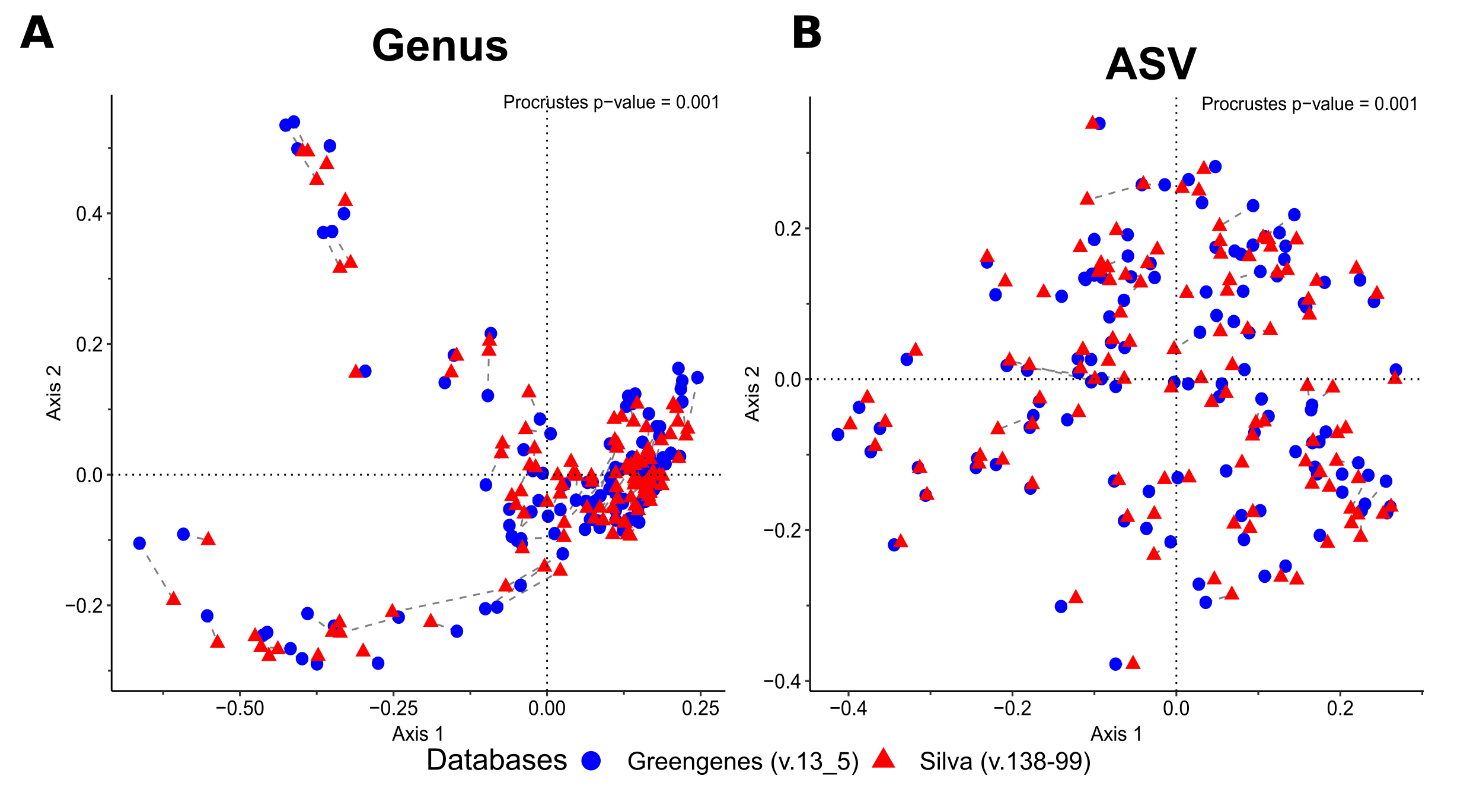


**2.1 Table 1: Key functions to import QIIME2 data into R and calculate basic alpha and beta diversity metrics.**

| **Function name** | **Short description** | **Package** |
| --- | --- | --- |
| *qza_to_phyloseq* | Generates a phyloseq object from QIIME2 artifacts. | Qiime2R |
| *Tax_glom* | Agglomerates phyloseq objects for a given taxonomic level. | Phyloseq |
| *distance* | Calculates a distance matrix for a given phyloseq object | Phyloseq |
| *ordinate* | Wraps several commonly used ordination methods using the distance object matrix computed by the distance function | Phyloseq |
| *plot_richness* | Computes several alpha-diversity metrics and returns a ggplot plotting object | Phyloseq |

**2.2 Table 2: Pairwise post-hoc comparisons of microbial genera relative abundances across 16S rRNA regions in mock samples**

This table presents the results of a post-hoc comparison test examining differences in relative abundances of microbial genera across various 16S rRNA regions (including the Zymo reference dataset, Ion Reporter [IR], and individual regions V2–V9). Each row compares two genera (listed under group1 and group2) within a specific region. The statistic column reports the test statistic from the pairwise comparison. The p and p.adj columns show the unadjusted and multiple-comparison-adjusted p-values, respectively, where p.adj.signif provides an indication of statistical significance (“ns” = not significant).

| region | group1 | group2 | statistic | p | p.adj | p.adj.signif |
| --- | --- | --- | --- | --- | --- | --- |
| Zymo | Bacillus | Cronobacter | -1.67705 | 0.093533 | 0.26306 | ns |
| Zymo | Bacillus | Klebsiella | -1.67705 | 0.093533 | 0.26306 | ns |
| Zymo | Cronobacter | Enterococcus | 1.677051 | 0.093533 | 0.26306 | ns |
| Zymo | Cronobacter | Escherichia | 1.677051 | 0.093533 | 0.26306 | ns |
| Zymo | Cronobacter | Lactobacillus | 1.677051 | 0.093533 | 0.26306 | ns |
| Zymo | Cronobacter | Listeria | 1.677051 | 0.093533 | 0.26306 | ns |
| Zymo | Cronobacter | Pseudomonas | 1.677051 | 0.093533 | 0.26306 | ns |
| Zymo | Cronobacter | Salmonella | 1.677051 | 0.093533 | 0.26306 | ns |
| Zymo | Cronobacter | Staphylococcus | 1.677051 | 0.093533 | 0.26306 | ns |
| Zymo | Enterococcus | Klebsiella | -1.67705 | 0.093533 | 0.26306 | ns |
| Zymo | Escherichia | Klebsiella | -1.67705 | 0.093533 | 0.26306 | ns |
| Zymo | Klebsiella | Lactobacillus | 1.677051 | 0.093533 | 0.26306 | ns |
| Zymo | Klebsiella | Listeria | 1.677051 | 0.093533 | 0.26306 | ns |
| Zymo | Klebsiella | Pseudomonas | 1.677051 | 0.093533 | 0.26306 | ns |
| Zymo | Klebsiella | Salmonella | 1.677051 | 0.093533 | 0.26306 | ns |
| Zymo | Klebsiella | Staphylococcus | 1.677051 | 0.093533 | 0.26306 | ns |
| Zymo | Bacillus | Enterococcus | 0 | 1 | 1 | ns |
| Zymo | Bacillus | Escherichia | 0 | 1 | 1 | ns |
| Zymo | Bacillus | Lactobacillus | 0 | 1 | 1 | ns |
| Zymo | Bacillus | Listeria | 0 | 1 | 1 | ns |
| Zymo | Bacillus | Pseudomonas | 0 | 1 | 1 | ns |
| Zymo | Bacillus | Salmonella | 0 | 1 | 1 | ns |
| Zymo | Bacillus | Staphylococcus | 0 | 1 | 1 | ns |
| Zymo | Cronobacter | Klebsiella | 0 | 1 | 1 | ns |
| Zymo | Enterococcus | Escherichia | 0 | 1 | 1 | ns |
| Zymo | Enterococcus | Lactobacillus | 0 | 1 | 1 | ns |
| Zymo | Enterococcus | Listeria | 0 | 1 | 1 | ns |
| Zymo | Enterococcus | Pseudomonas | 0 | 1 | 1 | ns |
| Zymo | Enterococcus | Salmonella | 0 | 1 | 1 | ns |
| Zymo | Enterococcus | Staphylococcus | 0 | 1 | 1 | ns |
| Zymo | Escherichia | Lactobacillus | 0 | 1 | 1 | ns |
| Zymo | Escherichia | Listeria | 0 | 1 | 1 | ns |
| Zymo | Escherichia | Pseudomonas | 0 | 1 | 1 | ns |
| Zymo | Escherichia | Salmonella | 0 | 1 | 1 | ns |
| Zymo | Escherichia | Staphylococcus | 0 | 1 | 1 | ns |
| Zymo | Lactobacillus | Listeria | 0 | 1 | 1 | ns |
| Zymo | Lactobacillus | Pseudomonas | 0 | 1 | 1 | ns |
| Zymo | Lactobacillus | Salmonella | 0 | 1 | 1 | ns |
| Zymo | Lactobacillus | Staphylococcus | 0 | 1 | 1 | ns |
| Zymo | Listeria | Pseudomonas | 0 | 1 | 1 | ns |
| Zymo | Listeria | Salmonella | 0 | 1 | 1 | ns |
| Zymo | Listeria | Staphylococcus | 0 | 1 | 1 | ns |
| Zymo | Pseudomonas | Salmonella | 0 | 1 | 1 | ns |
| Zymo | Pseudomonas | Staphylococcus | 0 | 1 | 1 | ns |
| Zymo | Salmonella | Staphylococcus | 0 | 1 | 1 | ns |
| IR | Escherichia | Listeria | 4.377138 | 1.20E-05 | 0.000271 | *** |
| IR | Klebsiella | Listeria | 4.377138 | 1.20E-05 | 0.000271 | *** |
| IR | Cronobacter | Listeria | 4.048852 | 5.15E-05 | 0.000769 | *** |
| IR | Escherichia | Lactobacillus | 3.928481 | 8.55E-05 | 0.000769 | *** |
| IR | Klebsiella | Lactobacillus | 3.928481 | 8.55E-05 | 0.000769 | *** |
| IR | Cronobacter | Lactobacillus | 3.600196 | 0.000318 | 0.002385 | ** |
| IR | Bacillus | Escherichia | -3.40322 | 0.000666 | 0.003746 | ** |
| IR | Bacillus | Klebsiella | -3.40322 | 0.000666 | 0.003746 | ** |
| IR | Listeria | Pseudomonas | -3.17342 | 0.001507 | 0.007533 | ** |
| IR | Bacillus | Cronobacter | -3.07494 | 0.002105 | 0.009475 | ** |
| IR | Escherichia | Staphylococcus | 2.954568 | 0.003131 | 0.011742 | * |
| IR | Klebsiella | Staphylococcus | 2.954568 | 0.003131 | 0.011742 | * |
| IR | Lactobacillus | Pseudomonas | -2.72477 | 0.006435 | 0.022274 | * |
| IR | Cronobacter | Staphylococcus | 2.626283 | 0.008632 | 0.025897 | * |
| IR | Listeria | Salmonella | -2.62628 | 0.008632 | 0.025897 | * |
| IR | Enterococcus | Escherichia | -2.298 | 0.021562 | 0.057076 | ns |
| IR | Enterococcus | Klebsiella | -2.298 | 0.021562 | 0.057076 | ns |
| IR | Bacillus | Pseudomonas | -2.19951 | 0.027842 | 0.069604 | ns |
| IR | Lactobacillus | Salmonella | -2.17763 | 0.029434 | 0.069712 | ns |
| IR | Enterococcus | Listeria | 2.07914 | 0.037604 | 0.08461 | ns |
| IR | Cronobacter | Enterococcus | 2.626283 | 0.008632 | 0.025897 | * |
| IR | Escherichia | Salmonella | 1.750855 | 0.079971 | 0.149945 | ns |
| IR | Klebsiella | Salmonella | 1.750855 | 0.079971 | 0.149945 | ns |
| IR | Pseudomonas | Staphylococcus | 1.750855 | 0.079971 | 0.149945 | ns |
| IR | Bacillus | Salmonella | -1.65237 | 0.098459 | 0.177227 | ns |
| IR | Enterococcus | Lactobacillus | 1.630484 | 0.102999 | 0.178268 | ns |
| IR | Cronobacter | Salmonella | -3.07494 | 0.002105 | 0.009475 | ** |
| IR | Listeria | Staphylococcus | -1.42257 | 0.154861 | 0.248884 | ns |
| IR | Escherichia | Pseudomonas | 1.203713 | 0.228701 | 0.331985 | ns |
| IR | Klebsiella | Pseudomonas | 1.203713 | 0.228701 | 0.331985 | ns |
| IR | Salmonella | Staphylococcus | 1.203713 | 0.228701 | 0.331985 | ns |
| IR | Bacillus | Enterococcus | -1.10523 | 0.269061 | 0.373405 | ns |
| IR | Enterococcus | Pseudomonas | -1.09428 | 0.27383 | 0.373405 | ns |
| IR | Bacillus | Listeria | 0.973913 | 0.3301 | 0.424414 | ns |
| IR | Lactobacillus | Staphylococcus | -0.97391 | 0.3301 | 0.424414 | ns |
| IR | Cronobacter | Pseudomonas | 2.626283 | 0.008632 | 0.025897 | * |
| IR | Enterococcus | Staphylococcus | 0.656571 | 0.511457 | 0.622042 | ns |
| IR | Enterococcus | Salmonella | -0.54714 | 0.584281 | 0.67417 | ns |
| IR | Pseudomonas | Salmonella | 0.547142 | 0.584281 | 0.67417 | ns |
| IR | Bacillus | Lactobacillus | 0.525257 | 0.599405 | 0.67433 | ns |
| IR | Bacillus | Staphylococcus | -0.44866 | 0.653679 | 0.700371 | ns |
| IR | Lactobacillus | Listeria | 0.448657 | 0.653679 | 0.700371 | ns |
| IR | Cronobacter | Escherichia | -0.32829 | 0.742696 | 0.759575 | ns |
| IR | Cronobacter | Klebsiella | -0.32829 | 0.742696 | 0.759575 | ns |
| IR | Escherichia | Klebsiella | 0 | 1 | 1 | ns |
| V2 | Cronobacter | Lactobacillus | 4.398147 | 1.09E-05 | 0.000164 | *** |
| V2 | Escherichia | Lactobacillus | 4.398147 | 1.09E-05 | 0.000164 | *** |
| V2 | Klebsiella | Lactobacillus | 4.398147 | 1.09E-05 | 0.000164 | *** |
| V2 | Cronobacter | Salmonella | 3.760416 | 0.00017 | 0.001272 | ** |
| V2 | Escherichia | Salmonella | 3.760416 | 0.00017 | 0.001272 | ** |
| V2 | Klebsiella | Salmonella | 3.760416 | 0.00017 | 0.001272 | ** |
| V2 | Bacillus | Cronobacter | -3.14468 | 0.001663 | 0.008314 | ** |
| V2 | Bacillus | Escherichia | -3.14468 | 0.001663 | 0.008314 | ** |
| V2 | Bacillus | Klebsiella | -3.14468 | 0.001663 | 0.008314 | ** |
| V2 | Lactobacillus | Pseudomonas | -3.01273 | 0.002589 | 0.011651 | * |
| V2 | Lactobacillus | Listeria | -2.96875 | 0.00299 | 0.012232 | * |
| V2 | Cronobacter | Staphylococcus | 2.770833 | 0.005591 | 0.017972 | * |
| V2 | Escherichia | Staphylococcus | 2.770833 | 0.005591 | 0.017972 | * |
| V2 | Klebsiella | Staphylococcus | 2.770833 | 0.005591 | 0.017972 | * |
| V2 | Cronobacter | Enterococcus | 2.353009 | 0.018622 | 0.046556 | * |
| V2 | Enterococcus | Escherichia | -2.35301 | 0.018622 | 0.046556 | * |
| V2 | Enterococcus | Klebsiella | -2.35301 | 0.018622 | 0.046556 | * |
| V2 | Pseudomonas | Salmonella | 2.374999 | 0.017549 | 0.046556 | * |
| V2 | Listeria | Salmonella | 2.331018 | 0.019752 | 0.046782 | * |
| V2 | Enterococcus | Lactobacillus | 2.045138 | 0.040841 | 0.091893 | ns |
| V2 | Bacillus | Pseudomonas | -1.75926 | 0.078534 | 0.168286 | ns |
| V2 | Bacillus | Listeria | -1.71528 | 0.086294 | 0.176511 | ns |
| V2 | Lactobacillus | Staphylococcus | -1.62731 | 0.10367 | 0.202833 | ns |
| V2 | Cronobacter | Listeria | 1.429398 | 0.15289 | 0.240859 | ns |
| V2 | Cronobacter | Pseudomonas | 1.385416 | 0.165925 | 0.240859 | ns |
| V2 | Enterococcus | Salmonella | 1.407407 | 0.159307 | 0.240859 | ns |
| V2 | Escherichia | Listeria | 1.429398 | 0.15289 | 0.240859 | ns |
| V2 | Escherichia | Pseudomonas | 1.385416 | 0.165925 | 0.240859 | ns |
| V2 | Klebsiella | Listeria | 1.429398 | 0.15289 | 0.240859 | ns |
| V2 | Klebsiella | Pseudomonas | 1.385416 | 0.165925 | 0.240859 | ns |
| V2 | Pseudomonas | Staphylococcus | 1.385416 | 0.165925 | 0.240859 | ns |
| V2 | Listeria | Staphylococcus | 1.341435 | 0.179779 | 0.252815 | ns |
| V2 | Bacillus | Lactobacillus | 1.253472 | 0.210034 | 0.28641 | ns |
| V2 | Salmonella | Staphylococcus | -0.98958 | 0.322378 | 0.426677 | ns |
| V2 | Enterococcus | Pseudomonas | -0.96759 | 0.333248 | 0.428462 | ns |
| V2 | Enterococcus | Listeria | -0.92361 | 0.355689 | 0.444611 | ns |
| V2 | Bacillus | Enterococcus | -0.79167 | 0.428555 | 0.521216 | ns |
| V2 | Lactobacillus | Salmonella | -0.63773 | 0.523649 | 0.62011 | ns |
| V2 | Bacillus | Salmonella | 0.615741 | 0.538066 | 0.620845 | ns |
| V2 | Enterococcus | Staphylococcus | 0.417824 | 0.676076 | 0.760585 | ns |
| V2 | Bacillus | Staphylococcus | -0.37384 | 0.708522 | 0.777646 | ns |
| V2 | Cronobacter | Escherichia | 0 | 1 | 1 | ns |
| V2 | Cronobacter | Klebsiella | 0 | 1 | 1 | ns |
| V2 | Escherichia | Klebsiella | 0 | 1 | 1 | ns |
| V2 | Listeria | Pseudomonas | -0.04398 | 0.964919 | 1 | ns |
| V2-9 | Cronobacter | Listeria | 4.519246 | 6.21E-06 | 0.00014 | *** |
| V2-9 | Escherichia | Listeria | 4.519246 | 6.21E-06 | 0.00014 | *** |
| V2-9 | Cronobacter | Lactobacillus | 3.974759 | 7.05E-05 | 0.000793 | *** |
| V2-9 | Escherichia | Lactobacillus | 3.974759 | 7.05E-05 | 0.000793 | *** |
| V2-9 | Bacillus | Cronobacter | -3.75696 | 0.000172 | 0.00129 | ** |
| V2-9 | Bacillus | Escherichia | -3.75696 | 0.000172 | 0.00129 | ** |
| V2-9 | Klebsiella | Listeria | 3.702515 | 0.000213 | 0.001372 | ** |
| V2-9 | Klebsiella | Lactobacillus | 3.158027 | 0.001588 | 0.007942 | ** |
| V2-9 | Listeria | Pseudomonas | -3.15803 | 0.001588 | 0.007942 | ** |
| V2-9 | Cronobacter | Staphylococcus | 2.994681 | 0.002747 | 0.011239 | * |
| V2-9 | Escherichia | Staphylococcus | 2.994681 | 0.002747 | 0.011239 | * |
| V2-9 | Bacillus | Klebsiella | -2.94023 | 0.00328 | 0.012299 | * |
| V2-9 | Lactobacillus | Pseudomonas | -2.61354 | 0.008961 | 0.028803 | * |
| V2-9 | Listeria | Salmonella | -2.61354 | 0.008961 | 0.028803 | * |
| V2-9 | Cronobacter | Enterococcus | 2.450194 | 0.014278 | 0.040157 | * |
| V2-9 | Enterococcus | Escherichia | -2.45019 | 0.014278 | 0.040157 | * |
| V2-9 | Bacillus | Pseudomonas | -2.39574 | 0.016587 | 0.043906 | * |
| V2-9 | Klebsiella | Staphylococcus | -3.15803 | 0.001588 | 0.007942 | ** |
| V2-9 | Enterococcus | Listeria | 2.069052 | 0.038541 | 0.086718 | ns |
| V2-9 | Lactobacillus | Salmonella | -2.06905 | 0.038541 | 0.086718 | ns |
| V2-9 | Cronobacter | Salmonella | 2.994681 | 0.002747 | 0.011239 | * |
| V2-9 | Escherichia | Salmonella | 1.905706 | 0.056688 | 0.115953 | ns |
| V2-9 | Bacillus | Salmonella | -1.85126 | 0.064133 | 0.125477 | ns |
| V2-9 | Enterococcus | Klebsiella | 2.994681 | 0.002747 | 0.011239 | * |
| V2-9 | Pseudomonas | Staphylococcus | 1.633462 | 0.102372 | 0.184269 | ns |
| V2-9 | Enterococcus | Lactobacillus | 1.524565 | 0.127368 | 0.212279 | ns |
| V2-9 | Listeria | Staphylococcus | -1.52456 | 0.127368 | 0.212279 | ns |
| V2-9 | Cronobacter | Pseudomonas | 3.974759 | 7.05E-05 | 0.000793 | *** |
| V2-9 | Escherichia | Pseudomonas | 1.361219 | 0.173445 | 0.269138 | ns |
| V2-9 | Bacillus | Enterococcus | -1.30677 | 0.191291 | 0.286936 | ns |
| V2-9 | Enterococcus | Pseudomonas | -1.08897 | 0.276165 | 0.376589 | ns |
| V2-9 | Klebsiella | Salmonella | 1.088975 | 0.276165 | 0.376589 | ns |
| V2-9 | Salmonella | Staphylococcus | 1.088975 | 0.276165 | 0.376589 | ns |
| V2-9 | Lactobacillus | Staphylococcus | -0.98008 | 0.327048 | 0.432858 | ns |
| V2-9 | Cronobacter | Klebsiella | 0.816731 | 0.414082 | 0.517603 | ns |
| V2-9 | Escherichia | Klebsiella | 0.816731 | 0.414082 | 0.517603 | ns |
| V2-9 | Bacillus | Listeria | 0.762282 | 0.445891 | 0.528029 | ns |
| V2-9 | Bacillus | Staphylococcus | -0.76228 | 0.445891 | 0.528029 | ns |
| V2-9 | Enterococcus | Salmonella | -0.54449 | 0.586106 | 0.613367 | ns |
| V2-9 | Enterococcus | Staphylococcus | 0.544487 | 0.586106 | 0.613367 | ns |
| V2-9 | Klebsiella | Pseudomonas | 0.544487 | 0.586106 | 0.613367 | ns |
| V2-9 | Lactobacillus | Listeria | 0.544487 | 0.586106 | 0.613367 | ns |
| V2-9 | Pseudomonas | Salmonella | 0.544487 | 0.586106 | 0.613367 | ns |
| V2-9 | Bacillus | Lactobacillus | 0.217795 | 0.827589 | 0.846398 | ns |
| V2-9 | Cronobacter | Escherichia | 0 | 1 | 1 | ns |
| V3 | Bacillus | Cronobacter | -4.05805 | 4.95E-05 | 0.000445 | *** |
| V3 | Bacillus | Escherichia | -4.05805 | 4.95E-05 | 0.000445 | *** |
| V3 | Bacillus | Klebsiella | -4.05805 | 4.95E-05 | 0.000445 | *** |
| V3 | Bacillus | Listeria | -4.05805 | 4.95E-05 | 0.000445 | *** |
| V3 | Bacillus | Staphylococcus | -4.05805 | 4.95E-05 | 0.000445 | *** |
| V3 | Cronobacter | Enterococcus | 3.03774 | 0.002384 | 0.010726 | * |
| V3 | Enterococcus | Escherichia | -3.03774 | 0.002384 | 0.010726 | * |
| V3 | Enterococcus | Klebsiella | -3.03774 | 0.002384 | 0.010726 | * |
| V3 | Enterococcus | Listeria | -3.03774 | 0.002384 | 0.010726 | * |
| V3 | Enterococcus | Staphylococcus | -3.03774 | 0.002384 | 0.010726 | * |
| V3 | Cronobacter | Lactobacillus | 2.875418 | 0.004035 | 0.012105 | * |
| V3 | Escherichia | Lactobacillus | 2.875418 | 0.004035 | 0.012105 | * |
| V3 | Klebsiella | Lactobacillus | 2.875418 | 0.004035 | 0.012105 | * |
| V3 | Lactobacillus | Listeria | -2.87542 | 0.004035 | 0.012105 | * |
| V3 | Lactobacillus | Staphylococcus | -2.87542 | 0.004035 | 0.012105 | * |
| V3 | Cronobacter | Salmonella | 2.782662 | 0.005391 | 0.012131 | * |
| V3 | Escherichia | Salmonella | 2.782662 | 0.005391 | 0.012131 | * |
| V3 | Klebsiella | Salmonella | 2.782662 | 0.005391 | 0.012131 | * |
| V3 | Listeria | Salmonella | 2.782662 | 0.005391 | 0.012131 | * |
| V3 | Salmonella | Staphylococcus | -2.78266 | 0.005391 | 0.012131 | * |
| V3 | Bacillus | Pseudomonas | -2.31889 | 0.020401 | 0.043717 | * |
| V3 | Cronobacter | Pseudomonas | 1.739164 | 0.082006 | 0.141933 | ns |
| V3 | Escherichia | Pseudomonas | 1.739164 | 0.082006 | 0.141933 | ns |
| V3 | Klebsiella | Pseudomonas | 1.739164 | 0.082006 | 0.141933 | ns |
| V3 | Listeria | Pseudomonas | 1.739164 | 0.082006 | 0.141933 | ns |
| V3 | Pseudomonas | Staphylococcus | -1.73916 | 0.082006 | 0.141933 | ns |
| V3 | Enterococcus | Pseudomonas | -1.29858 | 0.19409 | 0.323483 | ns |
| V3 | Bacillus | Salmonella | -1.27539 | 0.202172 | 0.32492 | ns |
| V3 | Bacillus | Lactobacillus | -1.18263 | 0.236955 | 0.367689 | ns |
| V3 | Lactobacillus | Pseudomonas | -1.13625 | 0.25585 | 0.383776 | ns |
| V3 | Pseudomonas | Salmonella | 1.043498 | 0.296718 | 0.430719 | ns |
| V3 | Bacillus | Enterococcus | -1.02031 | 0.307582 | 0.432537 | ns |
| V3 | Cronobacter | Escherichia | 0 | 1 | 1 | ns |
| V3 | Cronobacter | Klebsiella | 0 | 1 | 1 | ns |
| V3 | Cronobacter | Listeria | 0 | 1 | 1 | ns |
| V3 | Cronobacter | Staphylococcus | 0 | 1 | 1 | ns |
| V3 | Enterococcus | Lactobacillus | -0.16232 | 0.871052 | 1 | ns |
| V3 | Enterococcus | Salmonella | -0.25508 | 0.798663 | 1 | ns |
| V3 | Escherichia | Klebsiella | 0 | 1 | 1 | ns |
| V3 | Escherichia | Listeria | 0 | 1 | 1 | ns |
| V3 | Escherichia | Staphylococcus | 0 | 1 | 1 | ns |
| V3 | Klebsiella | Listeria | 0 | 1 | 1 | ns |
| V3 | Klebsiella | Staphylococcus | 0 | 1 | 1 | ns |
| V3 | Lactobacillus | Salmonella | -0.09276 | 0.926098 | 1 | ns |
| V3 | Listeria | Staphylococcus | 0 | 1 | 1 | ns |
| V4 | Cronobacter | Lactobacillus | 4.11422 | 3.88E-05 | 0.000437 | *** |
| V4 | Escherichia | Lactobacillus | 4.11422 | 3.88E-05 | 0.000437 | *** |
| V4 | Klebsiella | Lactobacillus | 4.11422 | 3.88E-05 | 0.000437 | *** |
| V4 | Lactobacillus | Listeria | -4.11422 | 3.88E-05 | 0.000437 | *** |
| V4 | Bacillus | Cronobacter | -3.73307 | 0.000189 | 0.001064 | ** |
| V4 | Bacillus | Escherichia | -3.73307 | 0.000189 | 0.001064 | ** |
| V4 | Bacillus | Klebsiella | -3.73307 | 0.000189 | 0.001064 | ** |
| V4 | Bacillus | Listeria | -3.73307 | 0.000189 | 0.001064 | ** |
| V4 | Cronobacter | Staphylococcus | 3.082862 | 0.00205 | 0.007688 | ** |
| V4 | Escherichia | Staphylococcus | 3.082862 | 0.00205 | 0.007688 | ** |
| V4 | Klebsiella | Staphylococcus | 3.082862 | 0.00205 | 0.007688 | ** |
| V4 | Listeria | Staphylococcus | 3.082862 | 0.00205 | 0.007688 | ** |
| V4 | Lactobacillus | Pseudomonas | -2.71292 | 0.006669 | 0.023086 | * |
| V4 | Cronobacter | Enterococcus | 2.432659 | 0.014988 | 0.039675 | * |
| V4 | Enterococcus | Escherichia | -2.43266 | 0.014988 | 0.039675 | * |
| V4 | Enterococcus | Klebsiella | -2.43266 | 0.014988 | 0.039675 | * |
| V4 | Enterococcus | Listeria | -2.43266 | 0.014988 | 0.039675 | * |
| V4 | Bacillus | Pseudomonas | -2.33176 | 0.019713 | 0.049283 | * |
| V4 | Cronobacter | Salmonella | 2.051505 | 0.040218 | 0.078687 | ns |
| V4 | Escherichia | Salmonella | 2.051505 | 0.040218 | 0.078687 | ns |
| V4 | Klebsiella | Salmonella | 2.051505 | 0.040218 | 0.078687 | ns |
| V4 | Lactobacillus | Salmonella | -2.06272 | 0.03914 | 0.078687 | ns |
| V4 | Listeria | Salmonella | 2.051505 | 0.040218 | 0.078687 | ns |
| V4 | Bacillus | Salmonella | -1.68156 | 0.092654 | 0.160363 | ns |
| V4 | Enterococcus | Lactobacillus | 1.681561 | 0.092654 | 0.160363 | ns |
| V4 | Pseudomonas | Staphylococcus | 1.681561 | 0.092654 | 0.160363 | ns |
| V4 | Cronobacter | Pseudomonas | 1.401301 | 0.161124 | 0.241686 | ns |
| V4 | Escherichia | Pseudomonas | 1.401301 | 0.161124 | 0.241686 | ns |
| V4 | Klebsiella | Pseudomonas | 1.401301 | 0.161124 | 0.241686 | ns |
| V4 | Listeria | Pseudomonas | 1.401301 | 0.161124 | 0.241686 | ns |
| V4 | Bacillus | Enterococcus | -1.30041 | 0.193461 | 0.280831 | ns |
| V4 | Enterococcus | Pseudomonas | -1.03136 | 0.302373 | 0.4002 | ns |
| V4 | Lactobacillus | Staphylococcus | -1.03136 | 0.302373 | 0.4002 | ns |
| V4 | Salmonella | Staphylococcus | 1.031358 | 0.302373 | 0.4002 | ns |
| V4 | Bacillus | Staphylococcus | -0.6502 | 0.515561 | 0.627033 | ns |
| V4 | Enterococcus | Staphylococcus | 0.650204 | 0.515561 | 0.627033 | ns |
| V4 | Pseudomonas | Salmonella | 0.650204 | 0.515561 | 0.627033 | ns |
| V4 | Bacillus | Lactobacillus | 0.381154 | 0.703089 | 0.811257 | ns |
| V4 | Enterococcus | Salmonella | -0.38115 | 0.703089 | 0.811257 | ns |
| V4 | Cronobacter | Escherichia | 0 | 1 | 1 | ns |
| V4 | Cronobacter | Klebsiella | 0 | 1 | 1 | ns |
| V4 | Cronobacter | Listeria | 0 | 1 | 1 | ns |
| V4 | Escherichia | Klebsiella | 0 | 1 | 1 | ns |
| V4 | Escherichia | Listeria | 0 | 1 | 1 | ns |
| V4 | Klebsiella | Listeria | 0 | 1 | 1 | ns |
| V67 | Cronobacter | Lactobacillus | 3.942105 | 8.08E-05 | 0.000727 | *** |
| V67 | Escherichia | Lactobacillus | 3.942105 | 8.08E-05 | 0.000727 | *** |
| V67 | Klebsiella | Lactobacillus | 3.942105 | 8.08E-05 | 0.000727 | *** |
| V67 | Lactobacillus | Listeria | -3.94211 | 8.08E-05 | 0.000727 | *** |
| V67 | Lactobacillus | Pseudomonas | -3.94211 | 8.08E-05 | 0.000727 | *** |
| V67 | Cronobacter | Salmonella | 3.594272 | 0.000325 | 0.001464 | ** |
| V67 | Escherichia | Salmonella | 3.594272 | 0.000325 | 0.001464 | ** |
| V67 | Klebsiella | Salmonella | 3.594272 | 0.000325 | 0.001464 | ** |
| V67 | Listeria | Salmonella | 3.594272 | 0.000325 | 0.001464 | ** |
| V67 | Pseudomonas | Salmonella | 3.594272 | 0.000325 | 0.001464 | ** |
| V67 | Bacillus | Cronobacter | -2.80585 | 0.005018 | 0.015055 | * |
| V67 | Bacillus | Escherichia | -2.80585 | 0.005018 | 0.015055 | * |
| V67 | Bacillus | Klebsiella | -2.80585 | 0.005018 | 0.015055 | * |
| V67 | Bacillus | Listeria | -2.80585 | 0.005018 | 0.015055 | * |
| V67 | Bacillus | Pseudomonas | -2.80585 | 0.005018 | 0.015055 | * |
| V67 | Cronobacter | Staphylococcus | 2.342074 | 0.019177 | 0.043148 | * |
| V67 | Escherichia | Staphylococcus | 2.342074 | 0.019177 | 0.043148 | * |
| V67 | Klebsiella | Staphylococcus | 2.342074 | 0.019177 | 0.043148 | * |
| V67 | Listeria | Staphylococcus | 2.342074 | 0.019177 | 0.043148 | * |
| V67 | Pseudomonas | Staphylococcus | 2.342074 | 0.019177 | 0.043148 | * |
| V67 | Enterococcus | Lactobacillus | 2.133374 | 0.032894 | 0.070487 | ns |
| V67 | Cronobacter | Enterococcus | 1.808731 | 0.070493 | 0.122007 | ns |
| V67 | Enterococcus | Escherichia | -1.80873 | 0.070493 | 0.122007 | ns |
| V67 | Enterococcus | Klebsiella | -1.80873 | 0.070493 | 0.122007 | ns |
| V67 | Enterococcus | Listeria | -1.80873 | 0.070493 | 0.122007 | ns |
| V67 | Enterococcus | Pseudomonas | -1.80873 | 0.070493 | 0.122007 | ns |
| V67 | Enterococcus | Salmonella | 1.785542 | 0.074173 | 0.123622 | ns |
| V67 | Lactobacillus | Staphylococcus | -1.60003 | 0.109592 | 0.17613 | ns |
| V67 | Salmonella | Staphylococcus | -1.2522 | 0.210498 | 0.326634 | ns |
| V67 | Bacillus | Lactobacillus | 1.136254 | 0.25585 | 0.383776 | ns |
| V67 | Bacillus | Enterococcus | -0.99712 | 0.318706 | 0.462638 | ns |
| V67 | Bacillus | Salmonella | 0.788421 | 0.43045 | 0.605321 | ns |
| V67 | Enterococcus | Staphylococcus | 0.533344 | 0.593796 | 0.809721 | ns |
| V67 | Bacillus | Staphylococcus | -0.46378 | 0.642807 | 0.850775 | ns |
| V67 | Lactobacillus | Salmonella | -0.34783 | 0.727966 | 0.935956 | ns |
| V67 | Cronobacter | Escherichia | 0 | 1 | 1 | ns |
| V67 | Cronobacter | Klebsiella | 0 | 1 | 1 | ns |
| V67 | Cronobacter | Listeria | 0 | 1 | 1 | ns |
| V67 | Cronobacter | Pseudomonas | 0 | 1 | 1 | ns |
| V67 | Escherichia | Klebsiella | 0 | 1 | 1 | ns |
| V67 | Escherichia | Listeria | 0 | 1 | 1 | ns |
| V67 | Escherichia | Pseudomonas | 0 | 1 | 1 | ns |
| V67 | Klebsiella | Listeria | 0 | 1 | 1 | ns |
| V67 | Klebsiella | Pseudomonas | 0 | 1 | 1 | ns |
| V67 | Listeria | Pseudomonas | 0 | 1 | 1 | ns |
| V8 | Cronobacter | Enterococcus | 3.687028 | 0.000227 | 0.001021 | ** |
| V8 | Cronobacter | Salmonella | 3.84935 | 0.000118 | 0.001021 | ** |
| V8 | Enterococcus | Escherichia | -3.68703 | 0.000227 | 0.001021 | ** |
| V8 | Enterococcus | Klebsiella | -3.68703 | 0.000227 | 0.001021 | ** |
| V8 | Enterococcus | Listeria | -3.68703 | 0.000227 | 0.001021 | ** |
| V8 | Enterococcus | Pseudomonas | -3.68703 | 0.000227 | 0.001021 | ** |
| V8 | Escherichia | Salmonella | 3.84935 | 0.000118 | 0.001021 | ** |
| V8 | Klebsiella | Salmonella | 3.84935 | 0.000118 | 0.001021 | ** |
| V8 | Listeria | Salmonella | 3.84935 | 0.000118 | 0.001021 | ** |
| V8 | Pseudomonas | Salmonella | 3.84935 | 0.000118 | 0.001021 | ** |
| V8 | Cronobacter | Staphylococcus | 2.875418 | 0.004035 | 0.012105 | * |
| V8 | Escherichia | Staphylococcus | 2.875418 | 0.004035 | 0.012105 | * |
| V8 | Klebsiella | Staphylococcus | 2.875418 | 0.004035 | 0.012105 | * |
| V8 | Listeria | Staphylococcus | 2.875418 | 0.004035 | 0.012105 | * |
| V8 | Pseudomonas | Staphylococcus | 2.875418 | 0.004035 | 0.012105 | * |
| V8 | Bacillus | Cronobacter | -2.34207 | 0.019177 | 0.043148 | * |
| V8 | Bacillus | Escherichia | -2.34207 | 0.019177 | 0.043148 | * |
| V8 | Bacillus | Klebsiella | -2.34207 | 0.019177 | 0.043148 | * |
| V8 | Bacillus | Listeria | -2.34207 | 0.019177 | 0.043148 | * |
| V8 | Bacillus | Pseudomonas | -2.34207 | 0.019177 | 0.043148 | * |
| V8 | Lactobacillus | Salmonella | 2.110186 | 0.034842 | 0.074662 | ns |
| V8 | Enterococcus | Lactobacillus | -1.94786 | 0.051431 | 0.1052 | ns |
| V8 | Cronobacter | Lactobacillus | 1.739164 | 0.082006 | 0.136677 | ns |
| V8 | Escherichia | Lactobacillus | 1.739164 | 0.082006 | 0.136677 | ns |
| V8 | Klebsiella | Lactobacillus | 1.739164 | 0.082006 | 0.136677 | ns |
| V8 | Lactobacillus | Listeria | -1.73916 | 0.082006 | 0.136677 | ns |
| V8 | Lactobacillus | Pseudomonas | -1.73916 | 0.082006 | 0.136677 | ns |
| V8 | Bacillus | Salmonella | 1.507275 | 0.13174 | 0.211725 | ns |
| V8 | Bacillus | Enterococcus | 1.344953 | 0.17864 | 0.2772 | ns |
| V8 | Lactobacillus | Staphylococcus | 1.136254 | 0.25585 | 0.383776 | ns |
| V8 | Salmonella | Staphylococcus | -0.97393 | 0.33009 | 0.479163 | ns |
| V8 | Enterococcus | Staphylococcus | -0.81161 | 0.417016 | 0.586428 | ns |
| V8 | Bacillus | Lactobacillus | -0.60291 | 0.546568 | 0.745321 | ns |
| V8 | Bacillus | Staphylococcus | 0.533344 | 0.593796 | 0.785906 | ns |
| V8 | Cronobacter | Escherichia | 0 | 1 | 1 | ns |
| V8 | Cronobacter | Klebsiella | 0 | 1 | 1 | ns |
| V8 | Cronobacter | Listeria | 0 | 1 | 1 | ns |
| V8 | Cronobacter | Pseudomonas | 0 | 1 | 1 | ns |
| V8 | Enterococcus | Salmonella | 0.162322 | 0.871052 | 1 | ns |
| V8 | Escherichia | Klebsiella | 0 | 1 | 1 | ns |
| V8 | Escherichia | Listeria | 0 | 1 | 1 | ns |
| V8 | Escherichia | Pseudomonas | 0 | 1 | 1 | ns |
| V8 | Klebsiella | Listeria | 0 | 1 | 1 | ns |
| V8 | Klebsiella | Pseudomonas | 0 | 1 | 1 | ns |
| V8 | Listeria | Pseudomonas | 0 | 1 | 1 | ns |
| V9 | Bacillus | Cronobacter | -2.23607 | 0.025347 | 0.126737 | ns |
| V9 | Bacillus | Enterococcus | -2.23607 | 0.025347 | 0.126737 | ns |
| V9 | Bacillus | Escherichia | -2.23607 | 0.025347 | 0.126737 | ns |
| V9 | Bacillus | Klebsiella | -2.23607 | 0.025347 | 0.126737 | ns |
| V9 | Bacillus | Lactobacillus | -2.23607 | 0.025347 | 0.126737 | ns |
| V9 | Bacillus | Listeria | -2.23607 | 0.025347 | 0.126737 | ns |
| V9 | Bacillus | Pseudomonas | -2.23607 | 0.025347 | 0.126737 | ns |
| V9 | Bacillus | Salmonella | -2.23607 | 0.025347 | 0.126737 | ns |
| V9 | Bacillus | Staphylococcus | -2.23607 | 0.025347 | 0.126737 | ns |
| V9 | Cronobacter | Enterococcus | 0 | 1 | 1 | ns |
| V9 | Cronobacter | Escherichia | 0 | 1 | 1 | ns |
| V9 | Cronobacter | Klebsiella | 0 | 1 | 1 | ns |
| V9 | Cronobacter | Lactobacillus | 0 | 1 | 1 | ns |
| V9 | Cronobacter | Listeria | 0 | 1 | 1 | ns |
| V9 | Cronobacter | Pseudomonas | 0 | 1 | 1 | ns |
| V9 | Cronobacter | Salmonella | 0 | 1 | 1 | ns |
| V9 | Cronobacter | Staphylococcus | 0 | 1 | 1 | ns |
| V9 | Enterococcus | Escherichia | 0 | 1 | 1 | ns |
| V9 | Enterococcus | Klebsiella | 0 | 1 | 1 | ns |
| V9 | Enterococcus | Lactobacillus | 0 | 1 | 1 | ns |
| V9 | Enterococcus | Listeria | 0 | 1 | 1 | ns |
| V9 | Enterococcus | Pseudomonas | 0 | 1 | 1 | ns |
| V9 | Enterococcus | Salmonella | 0 | 1 | 1 | ns |
| V9 | Enterococcus | Staphylococcus | 0 | 1 | 1 | ns |
| V9 | Escherichia | Klebsiella | 0 | 1 | 1 | ns |
| V9 | Escherichia | Lactobacillus | 0 | 1 | 1 | ns |
| V9 | Escherichia | Listeria | 0 | 1 | 1 | ns |
| V9 | Escherichia | Pseudomonas | 0 | 1 | 1 | ns |
| V9 | Escherichia | Salmonella | 0 | 1 | 1 | ns |
| V9 | Escherichia | Staphylococcus | 0 | 1 | 1 | ns |
| V9 | Klebsiella | Lactobacillus | 0 | 1 | 1 | ns |
| V9 | Klebsiella | Listeria | 0 | 1 | 1 | ns |
| V9 | Klebsiella | Pseudomonas | 0 | 1 | 1 | ns |
| V9 | Klebsiella | Salmonella | 0 | 1 | 1 | ns |
| V9 | Klebsiella | Staphylococcus | 0 | 1 | 1 | ns |
| V9 | Lactobacillus | Listeria | 0 | 1 | 1 | ns |
| V9 | Lactobacillus | Pseudomonas | 0 | 1 | 1 | ns |
| V9 | Lactobacillus | Salmonella | 0 | 1 | 1 | ns |
| V9 | Lactobacillus | Staphylococcus | 0 | 1 | 1 | ns |
| V9 | Listeria | Pseudomonas | 0 | 1 | 1 | ns |
| V9 | Listeria | Salmonella | 0 | 1 | 1 | ns |
| V9 | Listeria | Staphylococcus | 0 | 1 | 1 | ns |
| V9 | Pseudomonas | Salmonella | 0 | 1 | 1 | ns |
| V9 | Pseudomonas | Staphylococcus | 0 | 1 | 1 | ns |
| V9 | Salmonella | Staphylococcus | 0 | 1 | 1 | ns |

**2.3 Table 3:** **Pairwise post-hoc comparisons of alpha diversity indices among sequencing methods and 16S rRNA regions**

This table summarizes pairwise post-hoc comparisons of alpha diversity estimates across different 16S rRNA regions and analysis methods. Each row represents a comparison between two groups (listed under group1 and group2). Multiple alpha diversity metrics are included: Chao1, Observed (number of ASVs), Shannon, and Simpson. For each metric, both the raw p-value (p_value_X) and the adjusted p-value (BH, p.adj_X) are shown, where p-values have been corrected for multiple testing. The significance columns (sig_X) indicate whether any observed differences are statistically significant (“*”) or not (“ns”). These results aid in understanding how different sequencing approaches and targeted 16S regions affect the observed microbial community diversity.

| group1 | group2 | p_value_Chao1 | p_value_Observed | p_value_Shannon | p_value_Simpson | p.adj_Chao1 | p.adj_Observed | p.adj_Shannon | p.adj_Simpson | sig_Chao1 | sig_Observed | sig_Shannon | sig_Simpson |
| --- | --- | --- | --- | --- | --- | --- | --- | --- | --- | --- | --- | --- | --- |
| IR | Zymo | 0.505 | 0.505 | 0.242 | 0.242 | 0.513 | 0.513 | 0.255 | 0.255 | ns | ns | ns | ns |
| V2 | Zymo | 0.074 | 0.074 | 0.242 | 0.242 | 0.101 | 0.101 | 0.255 | 0.255 | ns | ns | ns | ns |
| V2 | IR | 0.177 | 0.177 | 0.242 | 0.012 | 0.209 | 0.209 | 0.255 | 0.020 | ns | ns | ns | * |
| V2-9 | IR | 0.067 | 0.067 | 0.242 | 0.242 | 0.101 | 0.101 | 0.255 | 0.255 | ns | ns | ns | ns |
| V2-9 | V2 | 0.004 | 0.004 | 0.144 | 0.403 | 0.020 | 0.020 | 0.176 | 0.419 | * | * | ns | ns |
| V3 | Zymo | 0.074 | 0.074 | 0.242 | 0.242 | 0.101 | 0.101 | 0.255 | 0.255 | ns | ns | ns | ns |
| V3 | IR | 0.007 | 0.007 | 0.012 | 0.012 | 0.020 | 0.020 | 0.020 | 0.020 | * | * | * | * |
| V3 | V2 | 0.004 | 0.004 | 0.012 | 0.012 | 0.020 | 0.020 | 0.020 | 0.020 | * | * | * | * |
| V3 | V2-9 | 0.004 | 0.004 | 0.012 | 0.012 | 0.020 | 0.020 | 0.020 | 0.020 | * | * | * | * |
| V4 | Zymo | 0.074 | 0.074 | 0.242 | 0.242 | 0.101 | 0.101 | 0.255 | 0.255 | ns | ns | ns | ns |
| V4 | IR | 0.007 | 0.007 | 0.012 | 0.012 | 0.020 | 0.020 | 0.020 | 0.020 | * | * | * | * |
| V4 | V2 | 0.004 | 0.004 | 0.012 | 0.012 | 0.020 | 0.020 | 0.020 | 0.020 | * | * | * | * |
| V4 | V2-9 | 0.004 | 0.004 | 0.012 | 0.012 | 0.020 | 0.020 | 0.020 | 0.020 | * | * | * | * |
| V4 | V3 | 0.004 | 0.004 | 0.095 | 0.296 | 0.020 | 0.020 | 0.125 | 0.310 | * | * | ns | ns |
| V67 | Zymo | 0.074 | 0.074 | 0.242 | 0.242 | 0.101 | 0.101 | 0.255 | 0.255 | ns | ns | ns | ns |
| V67 | IR | 0.007 | 0.007 | 0.012 | 0.012 | 0.020 | 0.020 | 0.020 | 0.020 | * | * | * | * |
| V67 | V2 | 0.004 | 0.004 | 0.012 | 0.012 | 0.020 | 0.020 | 0.020 | 0.020 | * | * | * | * |
| V67 | V2-9 | 0.004 | 0.004 | 0.012 | 0.012 | 0.020 | 0.020 | 0.020 | 0.020 | * | * | * | * |
| V67 | V4 | 0.004 | 0.004 | 0.095 | 0.095 | 0.020 | 0.020 | 0.125 | 0.125 | * | * | ns | ns |
| V8 | Zymo | 0.074 | 0.074 | 0.242 | 0.242 | 0.101 | 0.101 | 0.255 | 0.255 | ns | ns | ns | ns |
| V8 | IR | 0.007 | 0.007 | 0.012 | 0.012 | 0.020 | 0.020 | 0.020 | 0.020 | * | * | * | * |
| V8 | V2 | 0.004 | 0.004 | 0.012 | 0.012 | 0.020 | 0.020 | 0.020 | 0.020 | * | * | * | * |
| V8 | V2-9 | 0.004 | 0.004 | 0.012 | 0.012 | 0.020 | 0.020 | 0.020 | 0.020 | * | * | * | * |
| V8 | V4 | 0.004 | 0.004 | 0.012 | 0.022 | 0.020 | 0.020 | 0.020 | 0.035 | * | * | * | * |
| V9 | Zymo | 0.166 | 0.166 | 0.074 | 0.488 | 0.200 | 0.200 | 0.101 | 0.503 | ns | ns | ns | ns |
| V9 | IR | 0.009 | 0.009 | 0.007 | 0.131 | 0.020 | 0.020 | 0.020 | 0.162 | * | * | * | ns |
| V9 | V2 | 0.006 | 0.006 | 0.007 | 0.131 | 0.020 | 0.020 | 0.020 | 0.162 | * | * | * | ns |
| V9 | V2-9 | 0.006 | 0.006 | 0.007 | 0.131 | 0.020 | 0.020 | 0.020 | 0.162 | * | * | * | ns |
| V9 | V3 | 0.006 | 0.006 | 0.007 | 0.131 | 0.020 | 0.020 | 0.020 | 0.162 | * | * | * | ns |
| V9 | V4 | 0.006 | 0.006 | 0.007 | 0.131 | 0.020 | 0.020 | 0.020 | 0.162 | * | * | * | ns |
| V9 | V67 | 0.006 | 0.006 | 0.007 | 0.131 | 0.020 | 0.020 | 0.020 | 0.162 | * | * | * | ns |
| V9 | V8 | 0.006 | 0.006 | 0.007 | 0.131 | 0.020 | 0.020 | 0.020 | 0.162 | * | * | * | ns |
| V2-9 | Zymo | NA | NA | 0.242 | 0.242 | NA | NA | 0.255 | 0.255 | NA | NA | ns | ns |
| V67 | V3 | NA | NA | 0.531 | 0.531 | NA | NA | 0.531 | 0.531 | NA | NA | ns | ns |
| V8 | V3 | NA | NA | 0.012 | 0.022 | NA | NA | 0.020 | 0.035 | NA | NA | * | * |
| V8 | V67 | NA | NA | 0.060 | 0.095 | NA | NA | 0.095 | 0.125 | NA | NA | ns | ns |

**2.4 Table 4: PERMANOVA results for microbial community structure across 16S rRNA regions in mock samples**
This table presents the results of a Permutational Multivariate Analysis of Variance (PERMANOVA) test conducted to compare microbial community structures among different 16S rRNA regions and theoretical community assemblages. Each row corresponds to a specific pairwise comparison (noted in **DFName** and **RowNames**).

- **Df:** Degrees of freedom for each test.
- **SumOfSqs:** The sum of squared distances, indicating the amount of variation attributed to the factor under consideration.
- **R2:** Proportion of the total variation explained by the factor.
- **F:** F-statistic derived from the ratio of explained to unexplained variation.
- **Pr(>F):** The p-value associated with the F-statistic, indicating the probability of observing these results under the null hypothesis.

Comparisons with lower p-values (e.g., <0.05) suggest significant differences in community composition attributed to the tested factor (e.g., differences between specific regions or theoretical and observed communities).

| Df | SumOfSqs | R2 | F | Pr(>F) | RowNames | DFName |
| --- | --- | --- | --- | --- | --- | --- |
| 1 | 0.00 | 0.18 | 1.70 | 0.342 | region | V2-9_vs_IR |
| 1 | 0.04 | 0.80 | 15.95 | 0.167 | region | V2_vs_Zymo |
| 1 | 0.04 | 0.89 | 32.27 | 0.167 | region | V2-9_vs_Zymo |
| 1 | 0.06 | 0.88 | 30.47 | 0.167 | region | IR_vs_Zymo |
| 1 | 0.08 | 0.84 | 42.90 | 0.012 | region | V2_vs_V2-9 |
| 1 | 0.10 | 0.85 | 47.17 | 0.005 | region | V2_vs_IR |
| 1 | 0.11 | 0.72 | 20.96 | 0.007 | region | V2_vs_V67 |
| 1 | 0.11 | 0.68 | 16.83 | 0.014 | region | V4_vs_V67 |
| 1 | 0.12 | 0.85 | 22.96 | 0.167 | region | V4_vs_Zymo |
| 1 | 0.13 | 0.81 | 33.85 | 0.004 | region | V2_vs_V4 |
| 1 | 0.14 | 0.90 | 35.71 | 0.167 | region | V3_vs_Zymo |
| 1 | 0.14 | 0.83 | 39.28 | 0.007 | region | V4_vs_IR |
| 1 | 0.14 | 0.84 | 42.91 | 0.009 | region | V4_vs_V2-9 |
| 1 | 0.14 | 0.82 | 17.92 | 0.167 | region | V67_vs_Zymo |
| 1 | 0.20 | 0.89 | 65.32 | 0.004 | region | V2_vs_V3 |
| 1 | 0.20 | 0.85 | 45.16 | 0.003 | region | V3_vs_V4 |
| 1 | 0.20 | 0.89 | 33.09 | 0.167 | region | V8_vs_Zymo |
| 1 | 0.27 | 0.87 | 55.85 | 0.015 | region | V67_vs_IR |
| 1 | 0.27 | 0.88 | 59.41 | 0.005 | region | V67_vs_V2-9 |
| 1 | 0.30 | 0.87 | 51.73 | 0.011 | region | V3_vs_V67 |
| 1 | 0.33 | 0.85 | 47.00 | 0.012 | region | V67_vs_V8 |
| 1 | 0.35 | 0.94 | 135.80 | 0.007 | region | V3_vs_V2-9 |
| 1 | 0.36 | 0.96 | 93.94 | 0.167 | region | V3_vs_V9 |
| 1 | 0.41 | 0.95 | 144.97 | 0.008 | region | V3_vs_IR |
| 1 | 0.45 | 0.96 | 86.85 | 0.167 | region | V4_vs_V9 |
| 1 | 0.46 | 0.93 | 107.41 | 0.013 | region | V2_vs_V8 |
| 1 | 0.55 | 0.95 | 70.60 | 0.167 | region | V67_vs_V9 |
| 1 | 0.56 | 0.99 | 411.65 | 0.167 | region | V9_vs_V2-9 |
| 1 | 0.57 | 0.99 | 307.08 | 0.167 | region | V9_vs_IR |
| 1 | 0.60 | 0.98 | 262.42 | 0.167 | region | V2_vs_V9 |
| 1 | 0.61 | 0.94 | 122.33 | 0.005 | region | V3_vs_V8 |
| 1 | 0.62 | 0.93 | 109.84 | 0.007 | region | V4_vs_V8 |
| 1 | 0.65 | 0.95 | 161.87 | 0.009 | region | V8_vs_IR |
| 1 | 0.65 | 0.96 | 172.39 | 0.004 | region | V8_vs_V2-9 |
| 1 | 0.69 | 0.97 | 111.16 | 0.167 | region | V8_vs_V9 |

**2.5 Table 5: Comparison of beta dispersion estimates for observed vs. theoretical mock community composition**

This table presents the estimated differences in beta dispersion between the observed microbial community data (across various 16S rRNA regions) and a theoretical reference community (denoted as “theoric”). Each point represents the mean difference (diff) in beta dispersion for a given region-region comparison, with vertical lines indicating the 95% confidence intervals (spanning lwr to upr). The p adj values show the statistical significance after correcting multiple comparisons. Differences that do not depart substantially from zero and large adjusted p-values suggest that the observed communities do not differ significantly in dispersion from theoretical expectation.

| Dfname | diff | lwr | upr | p adj |
| --- | --- | --- | --- | --- |
| IR-Zymo | 0.028 | -0.156 | 0.213 | 1.000 |
| V2-Zymo | 0.037 | -0.147 | 0.222 | 0.999 |
| V2-9-Zymo | 0.023 | -0.161 | 0.207 | 1.000 |
| V3-Zymo | 0.046 | -0.138 | 0.231 | 0.994 |
| V4-Zymo | 0.039 | -0.145 | 0.223 | 0.998 |
| V67-Zymo | 0.051 | -0.134 | 0.235 | 0.990 |
| V8-Zymo | 0.051 | -0.133 | 0.236 | 0.989 |
| V9-Zymo | 0.000 | -0.238 | 0.238 | 1.000 |
| V2-IR | 0.009 | -0.097 | 0.115 | 1.000 |
| V2-9-IR | -0.006 | -0.112 | 0.101 | 1.000 |
| V3-IR | 0.018 | -0.088 | 0.124 | 1.000 |
| V4-IR | 0.010 | -0.096 | 0.117 | 1.000 |
| V67-IR | 0.022 | -0.084 | 0.129 | 0.998 |
| V8-IR | 0.023 | -0.083 | 0.129 | 0.998 |
| V9-IR | -0.028 | -0.213 | 0.156 | 1.000 |
| V2-9-V2 | -0.014 | -0.121 | 0.092 | 1.000 |
| V3-V2 | 0.009 | -0.097 | 0.115 | 1.000 |
| V4-V2 | 0.001 | -0.105 | 0.108 | 1.000 |
| V67-V2 | 0.013 | -0.093 | 0.120 | 1.000 |
| V8-V2 | 0.014 | -0.092 | 0.120 | 1.000 |
| V9-V2 | -0.037 | -0.222 | 0.147 | 0.999 |
| V3-V2-9 | 0.024 | -0.083 | 0.130 | 0.998 |
| V4-V2-9 | 0.016 | -0.090 | 0.122 | 1.000 |
| V67-V2-9 | 0.028 | -0.079 | 0.134 | 0.993 |
| V8-V2-9 | 0.029 | -0.078 | 0.135 | 0.991 |
| V9-V2-9 | -0.023 | -0.207 | 0.161 | 1.000 |
| V4-V3 | -0.008 | -0.114 | 0.099 | 1.000 |
| V67-V3 | 0.004 | -0.102 | 0.111 | 1.000 |
| V8-V3 | 0.005 | -0.101 | 0.111 | 1.000 |
| V9-V3 | -0.046 | -0.231 | 0.138 | 0.994 |
| V67-V4 | 0.012 | -0.095 | 0.118 | 1.000 |
| V8-V4 | 0.013 | -0.094 | 0.119 | 1.000 |
| V9-V4 | -0.039 | -0.223 | 0.145 | 0.998 |
| V8-V67 | 0.001 | -0.106 | 0.107 | 1.000 |
| V9-V67 | -0.051 | -0.235 | 0.134 | 0.990 |
| V9-V8 | -0.051 | -0.236 | 0.133 | 0.989 |

**Table 6: PERMANOVA Analysis Results for Clinical Variables**

Table presenting the results of PERMANOVA analysis assessing the variation explained (R²) and associated p-values for different clinical variables in the dataset. The "Variation" column indicates the proportion of variation explained (R² %) by each variable, and the corresponding p-values are provided to assess the significance of these associations.

Formula used = *vegan::adonis2(Unifrac distance matrix ~ variable, data = metadata, permutations = 9999)*

| Clinical Variable | Variation (R^2^ %) | p-value |
| --- | --- | --- |
| Families | 55.48 | 0.0009 |
| Patient or Caregiver | 4.30 | 0.4833 |
| BMI | 9.55 | 0.1918 |
